# The ethics of data sharing and biobanking in health research

**DOI:** 10.12688/wellcomeopenres.16351.1

**Published:** 2020-11-16

**Authors:** Susan Bull, Niresh Bhagwandin

**Affiliations:** 1 The Ethox Centre and Wellcome Centre for Ethics and Humanities, University of Oxford, Oxford, UK; 2 South African Medical Research Council, Cape Town, South Africa

**Keywords:** Data sharing, biobanking, governance, global health, LMIC, ethics

## Abstract

The importance of data sharing and biobanking are increasingly being recognised in global health research. Such practices are perceived to have the potential to promote science by maximising the utility of data and samples. However, they also raise ethical challenges which can be exacerbated by existing disparities in power, infrastructure and capacity. The Global Forum on Bioethics in Research (GFBR) convened in Stellenbosch, South Africa in November 2018, to explore the ethics of data sharing and biobanking in health research. Ninety-five participants from 35 countries drew on case studies and their experiences with sharing in their discussion of issues relating to respecting research participants and communities, promoting equitable sharing, and international and national approaches to governing data sharing and biobanking. In this editorial we will briefly review insights relating to each of these three themes.

Increasing recognition of the need for collective action to address health priorities is driving a rapid expansion of data sharing and biobanking; and transforming knowledge production in health research. Against this background, multiple normative and empirical justifications for promoting and restricting the sharing of health data and samples have been proposed ^1, 2^. In 2018, a Global Forum on Bioethics in Research (GFBR) was convened to explore ethical issues associated with data sharing and biobanking in low- and middle-income country (LMIC) research ^3^. Ninety-five participants from 35 countries discussed three cross-cutting themes: respect for research participants and communities, promoting equity, and advancing good-governance ^4^.

## Respecting participants and communities

There is widespread recognition of the importance of respecting and protecting the interests of participants and communities during data sharing and biobanking. While effective protection of participants’ confidentiality and privacy is a foundational requirement, additional key considerations include appropriate models of seeking consent to sharing, meaningful engagement with participants and communities, and the importance of evaluating approaches to benefit sharing.

In reviewing models of seeking consent to biobanking and data sharing, GFBR participants emphasised the importance of ensuring that models of consent are contextually sensitive and appropriate. For example, there was consensus that broad consent can be an appropriate model for biobanks if a number of conditions relating to trustworthy governance, appropriate information provision and engagement, and benefit sharing (including knowledge sharing) are met. However, in some settings, regulatory requirements, a lack of familiarity with broad consent models, and participants’ concerns about the potential misuse of samples, highlight the importance of evaluating whether consent models providing greater participant control over specific research uses and feedback preferences should be implemented.

GFBR participants recognised the importance of implementing effective approaches to community and public engagement around data sharing and biobanking which go beyond information provision and consent facilitation. Prospective tensions between cultural sensitivities and potential scientific gains associated with sharing data and samples were discussed at length, including cultural beliefs which constrain the export of samples. Participants highlighted the importance of respectful and genuinely consultative approaches to discussing the rationale and potential value of data and sample sharing. Funders can support such activities by commissioning the development of evidence-based community engagement strategies and ensuring such activities are appropriately resourced.

In discussing respectful responses to participant and community interests, including benefit-sharing, GFBR participants agreed that research findings and knowledge produced should be communicated to research participants and their relevant communities in a cycle of engagement. It is additionally important to look broadly at what kinds of benefits may be generated by sharing data and samples, and the development of appropriate and accountable approaches to sharing benefits amongst researchers, communities and participants.

## Equity

Data and sample sharing takes place within an international global health community comprised of a heterogeneous community of stakeholders, with differing interests, remits, values, authority and access to resources. Concerns have arisen that international data-sharing mandates are insufficiently responsive to LMIC country perspectives, interests and contexts, and that the disproportionate availability of resources in wealthy institutions means that while high income countries (HICs) are well equipped to make use of data shared from LMICs, the reverse is less often true ^5^. In reflecting on historical examples of inequitable sharing and ‘helicopter research’, GFBR participants agreed that data-sharing and biobanking policies and practices should seek to reduce inequities. Care is needed to encourage data and sample sharing and re-use in a manner that is appropriately responsive to the interests of study participants and their communities; to the researchers sharing, accessing and analysing data and samples; and the broader public which stands to benefit from secondary analyses ^6^.

To be effective, approaches to promoting equitable data sharing should address multiple capacity building and recognition requirements; requiring a broad shift in research culture and financial and policy support from multiple stakeholders, including funders, research institutions, journals and governments. Priority areas to address include:

- Consideration of equity issues during the development, implementation and review of data sharing policies and processes;
- Development of mechanisms for the appropriate recognition of all intellectual contributions to the research process, including those of primary researchers and their teams;
- Alignment of academic recognition and promotion with data sharing mandates, so that meta-analyses, data-sharing and capacity building activities are rewarded appropriately;
- Investment in the human resources, infrastructure and collaborative relationships required to enhance efficient data curation and secondary data analysis capacity in LMIC settings;
- Investment in sustainable and inclusive platforms for complex data integration and analysis, particularly for priority health areas in LMICs;
- Capacity-building approaches which support effective and contextually-sensitive review and governance of data-sharing and biobanking activities.

## Governance

Alongside respecting participants/communities and equity, good governance is imperative to underscore trust. The development and implementation of fair, ethical and accountable governance systems are key to ensuring that data and biological samples are shared in a trustworthy manner. While open science and open data are increasingly becoming accepted norms of good research, in practice a spectrum of approaches to sharing samples and data have been adopted.

At present, many LMIC countries have limited regulatory and governance structures for biobanking and data sharing ^7^, and very few research institutions in these countries have formal data sharing policies. The case-studies and discussions at the GFBR meeting highlighted the importance of developing approaches to governing data sharing and biobanking which are appropriately tailored to context at national and institutional levels ^8^. Such systems should ensure that the interests of study participants and communities, data providers and data users, and the public are appropriately recognized and respected ^9^.

Understandably, there are several challenges in data sharing and the operation of biobanks, especially in LMICs. These include: understanding and responsibility for governance, lack of a legal framework and regulations, monitoring and enforcement, conflict of interest, exploitation, the remit of research ethics committees, maintenance of biobanks, technical abilities to govern, capacity and institutionalized ways of doing things that are difficult to change, lack of data expertise, long-term storage of personal health information and samples ^10^, capacity and technology issues, lack of harmonization and validation and cost in putting databases together.

Despite these numerous challenges, data sharing and biobanking can flourish if key elements of governance are in place:

- **Harmonisation of governance guidelines** – this calls for ongoing collaboration between members of the research community, partner organisations, participants and key stakeholders so that ethical codes are not breached when sharing data and transfer of biological samples locally and across borders.
- **Transparency and accountability** - researchers and interested parties should know where biological samples have come from and whether they were ethically obtained. In the same vein, biobanks and donors should know which research groups and organisations are using their samples, and the research they are supporting ^11^.
- **Community engagement/involvement** – this should be a formal component of a good governance system as it has implications not just for individuals but also for their families and communities. In some LMIC contexts, individuals often take decisions in consultation with family and community members ^12^.
- **Non-exploitation** - benefit sharing arising from the use of data and samples should not take the form of paternalistic tokenism. Exploitation can take many forms including HIC research collaborators and funders using the power differential to enforce their own views and policy imperatives on LMIC researchers.(Michael Pepper, personal communication, 2020)
- **Rational compliance** – policies and legal requirements for data sharing and biobanking should facilitate research, not impose unnecessary obligations on the research community.
- **Conflict of interest** – these need to be managed as in some cases those that establish the biobank are the users of the resource. GFBR participants noted that not allowing such access may impede some types of research.
- **Flexibility** – as research and technology advances, governance mechanisms should be flexible to accommodate these developments. For example, data access committees need to be established with specialized knowledge on biobank and data management.
- **External review** – regular reviews by independent bodies should be undertaken to ensure that data and samples are shared in a responsible manner and in keeping with conditions of access.

## Conclusion

Sharing health data and samples has the potential to improve our scientific understanding of health and disease, and inform improvements in healthcare and the health of populations. Increasing recognition of the need for collective action to address global health issues is driving a rapid expansion of multinational research activities incorporating biobanking and data sharing. Effective sharing requires a significant investment of resources to establish and maintain curation standards, methods and infrastructure, in addition to appropriate governance policies and processes. To effectively address global health needs, approaches to developing data and sample sharing platforms and processes should focus not just on maximising utility, but also recognise the varying priorities and values of a range of stakeholders. Participatory and procedurally fair processes are needed to develop equitable approaches to sharing which are responsive to the interests of study participants, communities, researchers, and national institutions involved in collecting and curating data, as well as the broader public which will potentially benefit from sharing.

## Data availability

No data are associated with this article.
